# *In situ* Investigations of the Formation Mechanism of Metastable *γ*‐BiPd Nanoparticles in Polyol Reductions

**DOI:** 10.1002/open.202300103

**Published:** 2023-12-13

**Authors:** Matthias Smuda, Noah Elsner, Jonas Ströh, Nicole Pienack, Rastko Radulovic, Azat Khadiev, Huayna Terraschke, Michael Ruck, Thomas Doert

**Affiliations:** ^1^ Faculty of Chemistry and Food Chemistry Technische Universität Dresden 01062 Dresden Germany; ^2^ Institute of Inorganic Chemistry Christian-Albrechts-Universität zu Kiel Max-Eyth-Str. 2 24118 Kiel Germany; ^3^ Deutsches Elektronen-Synchrotron DESY Notkestr. 85 22607 Hamburg Germany; ^4^ Max Planck Institute for Chemical Physics of Solids Nöthnizer Str. 40 01187 Dresden Germany

**Keywords:** γ-BiPd, formation mechanism, in-situ characterization, intermetallic nanoparticles, polyol process

## Abstract

Synthesizing intermetallic phases containing noble metals often poses a challenge as the melting points of noble metals often exceed the boiling point of bismuth (1560 °C). Reactions in the solid state generally circumvent this issue but are extremely time consuming. A convenient method to overcome these obstacles is the co‐reduction of metal salts in polyols, which can be performed within hours at moderate temperatures and even allows access to metastable phases. However, little attention has been paid to the formation mechanisms of intermetallic particles in polyol reductions. Identifying crucial reaction parameters and finding patterns are key factors to enable targeted syntheses and product design. Here, we chose metastable γ‐BiPd as an example to investigate the formation mechanism from mixtures of metal salts in ethylene glycol and to determine critical factors for phase formation. The reaction was also monitored by *in situ* X‐ray diffraction using synchrotron radiation. Products, intermediates and solutions were characterized by (in situ) X‐ray diffraction, electron microscopy, and UV‐Vis spectroscopy. In the first step of the reaction, elemental palladium precipitates. Increasing temperature induces the reduction of bismuth cations and the subsequent rapid incorporation of bismuth into the palladium cores, yielding the *γ*‐BiPd phase.

## Graphical abstract

The formation of *γ*‐BiPd in a polyol process has been monitored by X‐ray powder diffraction, light scattering, and *in situ* measurements of redox potential and pH value. Palladium nanoparticles are formed as primary reaction product followed by a successive reduction of bismuth cations and a diffusion‐controlled formation of the intermetallic target phase.

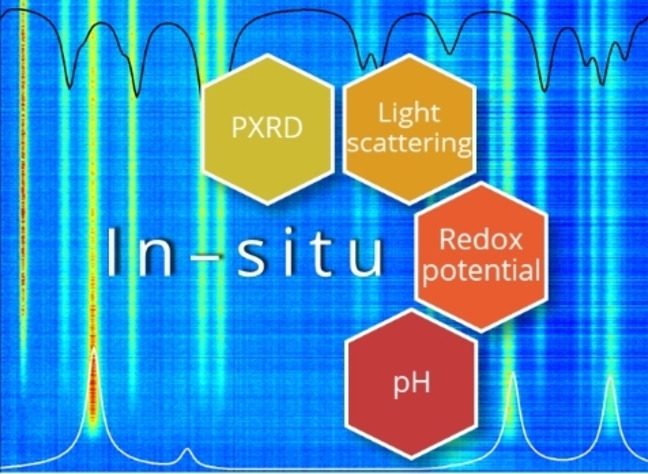

## Introduction

Intermetallic nanomaterials are intensively researched, as they combine the various materials properties of this huge compound class with a high specific surface area. They find application in numerous fields, such as heterogeneous catalysis,^[1]^ superconductors,^[2]^ hydrogen storage,^[3]^ or thermoelectrics.^[4]^ Especially, compounds containing bismuth and electron‐rich transition metals are tantalizing, as in many cases they are superconductive.^[5]^ However, the high melting points of noble metals, like 1555 °C for Pd, reach or surpass the boiling point of bismuth (1560 °C), making crystallization of compounds with predefined compositions from melts challenging.^[6]^ Moreover, considering the complexity of the Bi−Pd phase diagram, a single‐phase product from the melt would be hardly possible due to peritectic phase formation.^[7]^ Solid‐state syntheses can be performed to bypass these issues. However, such syntheses might take days or weeks at elevated temperatures and are, thus, time and energy consuming. Addressing metastable phases, such as *γ*‐BiPd, by high‐temperature synthesis is even more challenging. Furthermore, the obtained products are often agglomerates of particles or ingots, making subsequent processing in terms of size and shape control difficult. Ball milling or grinding can be used, but the structure can be damaged, influencing the particles’ properties, and these methods do not yield a homogeneous size distribution.

A convenient, time‐ and energy‐efficient method to obtain metallic and intermetallic particles is the polyol process, first introduced by Fievét.^[8]^ The polyalcohols (polyols) serve a threefold purpose. Due to their multiple hydroxyl groups, they possess good chelating properties, which leads to a similar solvation behavior as water, though some additional metal salts, which are insoluble in water, can be dissolved as well.^[9]^ Moreover, the insolubility and precipitation of products can be derived from their insolubility in water as well. The high boiling points enable the synthesis of crystalline particles and allow for an easier experimental setup, since the experiments can be performed at ambient pressure without the use of autoclaves. Second, the hydroxyl groups are able to stabilize the particles and impede agglomeration. Furthermore, by judiciously choosing the type of polyol and/or auxiliaries, for example, polyvinylpyrrolidone, the morphology, size, and even crystal structure of a polymorphous compound can be controlled.^[10]^ Third, and maybe more important the polyol is the reducing agent for the metal cations. By raising the reaction temperature, its reduction strength is increased and the reduction of various metals is enabled.^[11]^ Generally, noble metal cations, that is, those with a high positive standard potential, can be reduced at lower temperatures compared to cations of less noble metals, for example, Ni, Co, or Cu. The reductive power can be further increased by addition of a base to the reaction mixture due to deprotonation of the alcohol. According to *ab initio* calculations, the improved performance in the case of ethylene glycol (EG) is due to the glycolate monoanion HOC_2_H_4_O^−^, which exhibits an elevated HOMO, closing the gap to the LUMO of the metal cation.

Longer‐chain polyols can be utilized to achieve higher reaction temperatures, which is, however, accompanied by a decrease in reduction strength.^[12]^ To circumvent the natural limit posed by the boiling point and simultaneously exploit the reductive strength of short‐chain polyols, for example, EG or glycerol, a laboratory microwave with closed vessels can be used. In addition, the introduction of microwave heating offers multiple advantages over conventional heating methods, such as oil baths or heating mantles. The most important factor is the precise *in situ* control of the temperature profile, granting a high level of reproducibility. Furthermore, microwave radiation is more homogeneously distributed throughout the vessel, diminishing temperature gradients in the reactions mixture and restricting side reactions.^[13]^ In combination with the extreme heating rates which favor homogeneous nucleation, a uniform product can be consistently achieved, making this setup ideal for syntheses and mechanistic investigations thereof.^[14]^

Despite the (microwave‐assisted) polyol process’ well established position in the synthesis of intermetallic particles, mechanistic investigations were mainly performed on mono‐metallic reactions.^[15]^ A first comprehensive study on a more complex system was performed by Leonard and Schaak, elucidating the formation of AuCuSn_2_ particles in a modified polyol process.^[16]^ Lateron, Canaud et al. and Ying et al. reported on the formation of Ag_3_Sn and NiSn_5_ particles, respectively.^[17]^ Indeed, the understanding of reaction and formation mechanism is crucial for a targeted synthesis. Recently, an intriguing approach to reveal the formation of PtSn particles was conducted by the group of Guo, by using a sophisticated microfluidic system. After the identification of main steps in the formation process, accurate control of tube length and temperature enabled an optimized synthesis of PtSn chain‐like structures with good electrochemical performance in the ethanol oxidation reaction.^[18]^ The target‐oriented assembly of hollow Co/Cu structures and nanotubes and a fine‐tuning of particle size and morphology was also possible after the formation mechanism was understood. These results paved the way for designing various transition metal – noble metal nanostructures including some catalytically active species.^[19]^

In a small series of publications, we reported about the formation mechanisms of bismuth containing binary intermetallics by polyol reduction, namely BiNi, Bi_2_Ir, and Bi_2_Rh recently.^[20]^ Depending on the metal combination we found substantially diverse reaction mechanisms as well as different intermediates. The formation of BiNi particles follows a successive reduction of bismuth and nickel cations, where 2–10 nm sized particles encase the bismuth particle forming a self‐assembled Bi@Ni core‐shell structure. Subsequently, nickel diffuses into the bismuth core yielding Bi_3_Ni before the final product BiNi is obtained. In contrast, the hitherto unknown intermetallic suboxide Bi_4_Ir_2_O was obtained during formation of Bi_2_Ir as first intermediate *via* a partial co‐reduction Bi^3+^ and Ir^3+^ cations. The target compound is only obtained by further increase of the reaction temperature by complete reduction of the suboxide. In the case of *α*‐Bi_2_Rh, a co‐reduction of bismuth and rhodium cations yields BiRh particles. At higher temperatures, the residual bismuth cations are reduced, followed by a rapid diffusion into the BiRh particle, which results the final *α*‐Bi_2_Rh phase.

In this contribution, we expand our mechanistic studies towards the formation of a metastable binary phase, namely *γ*‐BiPd. *γ*‐BiPd had been prepared by polyol reduction before^[7b]^ and the low temperatures required to obtain *γ*‐BiPd make this phase also a suitable candidate for *in situ* studies by using different sensors directly in solution, see below. To ensure comparability with our previous works, we limit the investigations to ethylene glycol as solvent.

## Results and Discussion

The formation of *γ*‐BiPd particles was explored in a (microwave‐assisted) polyol process. Systematic *ex situ* experiments were performed by varying starting materials, reaction temperature, and pH value. Products, intermediates, and solutions were isolated and characterized by powder X‐ray diffraction (PXRD), scanning electron microscopy (SEM), energy‐dispersive X‐ray spectroscopy (EDS), and UV‐Vis spectroscopy. Additional reactions were performed in a glass reactor placed in a heating mantle (Figure S1) to enable *in situ* PXRD using synchrotron radiation as well as *in situ* measurements of temperature, redox potential, light scattering, and pH value.

### Reduction of Pd(OAc)_2_, PdCl_2_ and K_2_[PdBr_4_] in EG

**Figure 1:**
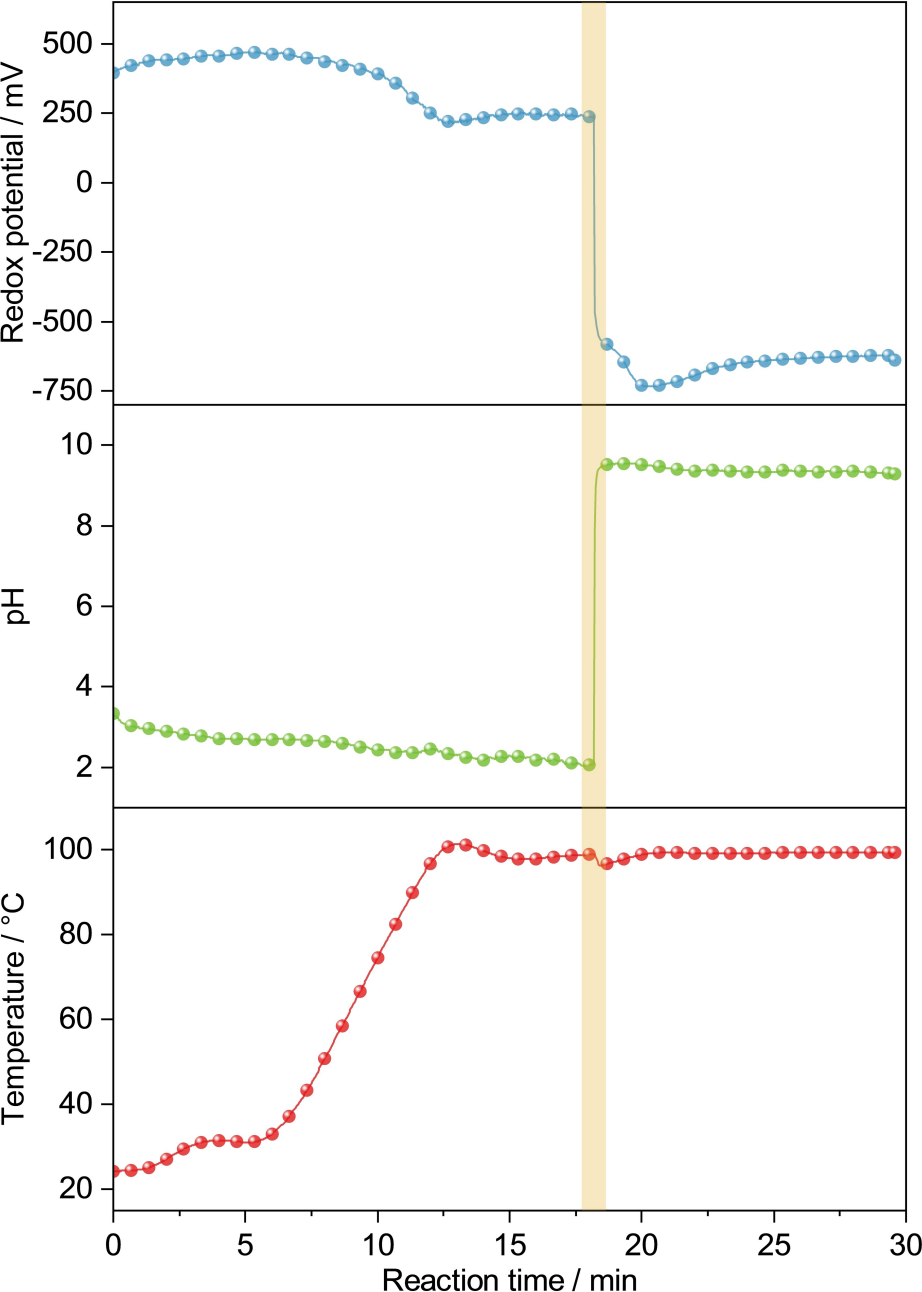
Course of the redox potential (Ag/AgCl reference) and pH upon heating and addition of KOH. The orange bar marks the addition of KOH.

To draw a complete picture, the reaction behavior of neat EG and each palladium precursor will be discussed briefly. At first, we investigated the interdependencies of pH, redox potential, and temperature in pure EG (Figure 1). Although the applied sensors are calibrated for aqueous solutions, the obtained data allows for a qualitative overview. Pure EG is weakly acidic at ambient temperature, and upon heating protolysis increases slightly. At the same time, the redox potential decreases, which indicates a rise in reduction strength. Increasing the pH by adding a KOH solution significantly enhances this effect (orange bar in Figure 1).

The reduction and solution chemistry of bismuth in polyols has been already discussed in previous works.[^20b^, ^20c^, ^21^] Although the tabulated values for standard potentials are given for aqueous solutions, they can be considered to approximately assess the feasibility of the reduction in polyol media (Eq. 1–3).^[22]^ Hence, palladium is more noble than bismuth, which leads to a higher temperature necessary for the reduction of bismuth cations.^[23]^

(1)

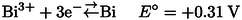



(2)

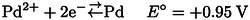



(3)

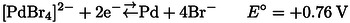




**Figure 2:**
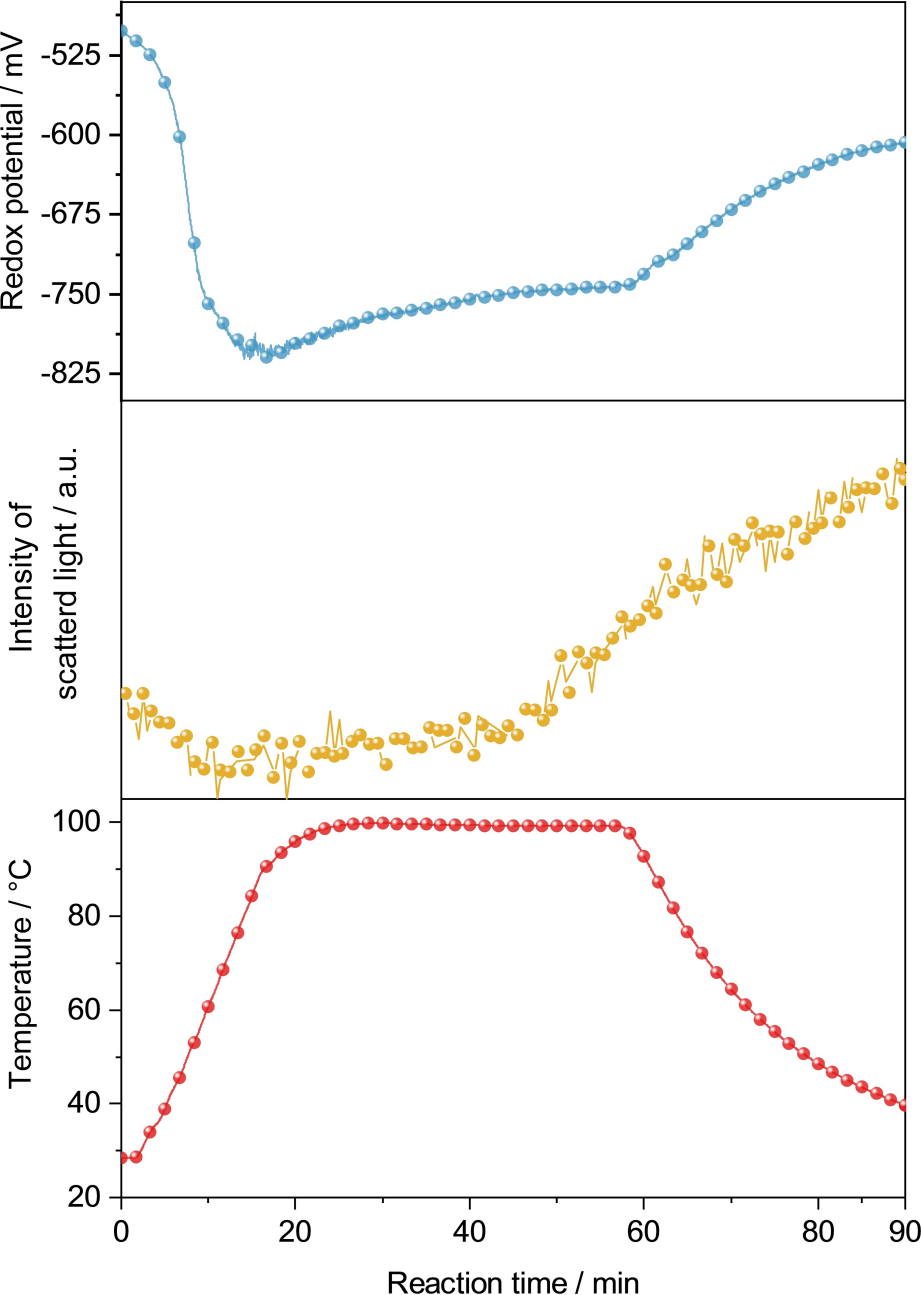
*In situ* measurements of redox potential (Ag/AgCl reference) and light scattering during the reduction of Pd(OAc)_2_ in alkaline EG.

Palladium acetate is an orange powder and insoluble in EG, thus, resulting in an orange dispersion upon mixing. Pd(OAc)_2_ is quickly reduced in neat EG at approx. 70 °C, yielding a black precipitate of palladium powder. Under alkaline conditions this conversion takes place at roughly 50 °C. This reduction was followed by *in situ* measurements of the redox potential of the reaction solution as well as measuring light scattering due to the Tyndall effect (Figure 2). The redox potential of the liquid phase, already being negative due to prior addition of KOH, decreases further with increasing temperature as observed for a neat EG solution. The intensity of the scattered light in the initial stage of reduction is slightly reduced, probably because the dispersion decreases slightly on Pd(OAc)_2_ when palladium nuclei are formed. Since noble metal salts tend to produce very small nanoparticles upon reduction (10–30 nm), the Tyndall effect is not as prominent. A prolonged reaction time, however, leads to agglomeration of the palladium particles, resulting in clusters large enough to be detectable by light scattering. Comparable observations were also made for the reduction of PdCl_2_ and K_2_[PdBr_4_] (Figure S2a&b).

The dark‐brown palladium(II) chloride is insoluble in EG at room temperature and yields a dark dispersion after sonication. Due to its strong Lewis acidic nature, the chloride ion is a strongly bound ligand that is not easily replaced by the polyol. Therefore, metal chlorides require substantially harsher reduction conditions, such as high pH or increased temperature.[^15a^, ^15d^] However, this effect is not as prevalent in the reduction reaction of PdCl_2_. The necessary reduction temperature only rises to approx. 75 °C for pure EG and can be lowered to 60 °C by adding KOH.

The last salt which was tested in this series is K_2_[PdBr_4_]. The dark‐red salt readily dissolves in EG yielding a reddish solution. A UV‐Vis spectrum displays distinct absorption bands at 525 nm and 415 nm accompanied by a strong absorbance in the UV region. (Figure S3) The addition of KOH, however, changes the color to a pale yellow. The absorption in the green region vanishes, while an absorption band at 365 nm appears. Most likely, the color change is caused by the formation of K_2_[Pd(OH)_4_], which can be reversed by addition of HBr solution. Owing to its lower standard potential, the reduction reaction requires a higher temperature (approx. 135 °C) than Pd(OAc)_2_ and PdCl_2_. Here, the addition of KOH has the strongest effect, lowering the reduction temperature to 50–70 °C.

### Reaction of Pd(OAc)_2_ and Bi(NO_3_)_3_ in EG

**Figure 3:**
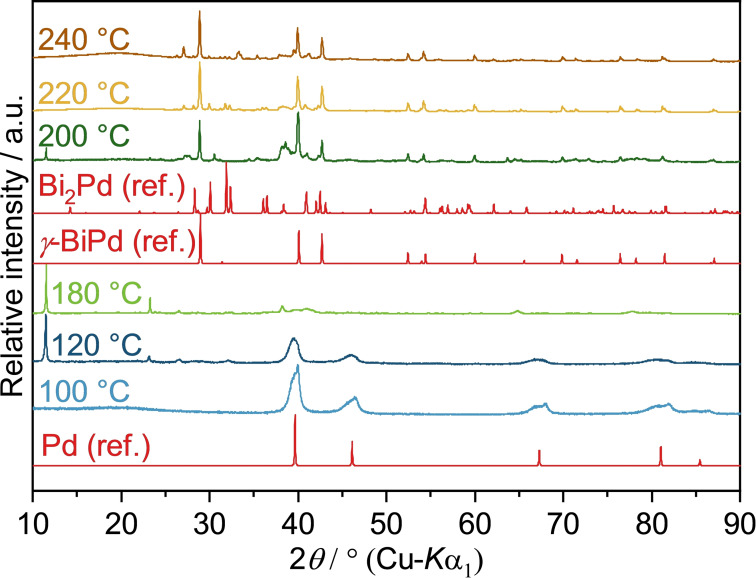
PXRD patterns of solid reaction products obtained from the reaction of Pd(OAc)_2_ and Bi(NO_3_)_3_ in neat EG at different temperatures after 10 min reaction time. Pd (ref.), *γ*‐BiPd (ref.) and Bi_2_Pd (ref.) are the calculated diffraction patterns based on the crystal structures of Pd (ICSD‐257579), *γ*‐BiPd (ICSD‐108171) and Bi_2_Pd (ICSD‐42565), respectively. The reflections at 11.5° and 23.3° found in the products obtained at 120 °C, 180 °C, and 200 °C, indicate the intermediate formation of a Bi glycolate.

In order to better separate time and temperature effects on the diffusion and reaction processes, experiments were performed with constant 10 minutes reaction time at selected temperatures. Between 60 to 100 °C, a solution of Pd(OAc)_2_ and Bi(NO_3_)_3_ (Pd^2+^ : Bi^3+^=1 : 1) in EG yielded a black precipitate which, according to PXRD and EDX, consists of elemental palladium (Figure 3). This is not surprising considering the large discrepancy in standard potentials between Bi^3+^ and Pd^2+^. Raising the temperature to 120 °C resulted in a mixture of palladium and a bismuth glycolate. Two sharp and intense reflection at 11.5° and 23.3° (2theta) coincide with the most intense peaks of a Bi glycolate which was also found in the reaction between Bi(NO_3_) and Rh_2_(OAc)_4_; its formula was assumed to be Bi_3_(C_2_H_4_O_2_)_4_(NO_3_).^[20c]^ At 200 °C, the glycolate is seemingly reduced to elemental bismuth, which is then available for the reaction with the palladium particles. The reaction between the metals occurs rapidly, which results in the dominance of the *γ*‐BiPd phase. However, the target compound is not obtained as a single‐phase product. Reflections of residual bismuth glycolate and traces of Bi_2_Pd can be identified, as well as a broad reflection at 38.5°, which could not be attributed to any known phase with certainty. Note that the reflections of different Bi−Pd compounds, for example *α*‐, *β*‐ and *γ*‐BiPd (ICSD‐54976, 56279 and 108171), Bi_2_Pd_5_ (ICSD‐58840), Bi_3_Pd_8_ (ICSD‐616947) or Bi_2_Pd (ICSD‐42565) overlap to a large extent so that an unambiguous assignment of individual side phases is not always possible. Although the reflections of the bismuth glycolate and the unknown phase disappear at higher temperatures, a phase pure product is not achieved, which might be due to minor stoichiometric errors or a too short reaction time.

Another remarkable feature is the change of the peak shape, especially the half‐width, of the Pd reflections. The peak widths generally indicate the formation of Pd nanoparticles. A very coarse estimation using the Scherrer equation yields an average particle size of ca. 80 nm for the samples obtained at 100 °C.^[24]^ At 120 °C the peak size is reduced to about 70 nm. This can be explained by the onset of Bi−Pd compound formation, which starts from the surface of the Pd primary particles.

**Figure 4:**
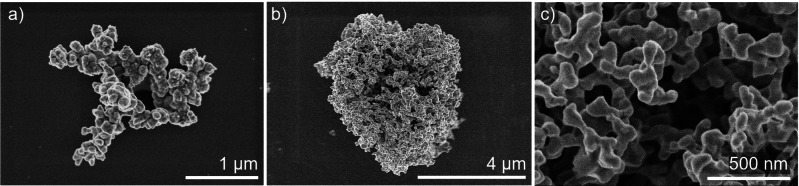
SEM images of particles obtained from the reaction of Pd(OAc)_2_ and Bi(NO_3_)_3_ in neat EG after 10 min at (a) 120 °C and (b)&(c) 220 °C.

Images of the isolated products recorded *via* SEM show mostly agglomerated particles throughout the reaction (Figure 4). At 120 °C, the image displays large structures of agglomerated particles with mostly quasi‐spherical morphology (Figure 4a). The product obtained at 220 °C, on the other hand, yielded porous agglomerates of particles with a diameter of approx. 50–150 nm (Figure 4b&c). Here, EDS resulted in a ratio of 46 : 53 (Bi : Pd), which corresponds to the desired ratio of 1 : 1.

**Figure 5:**
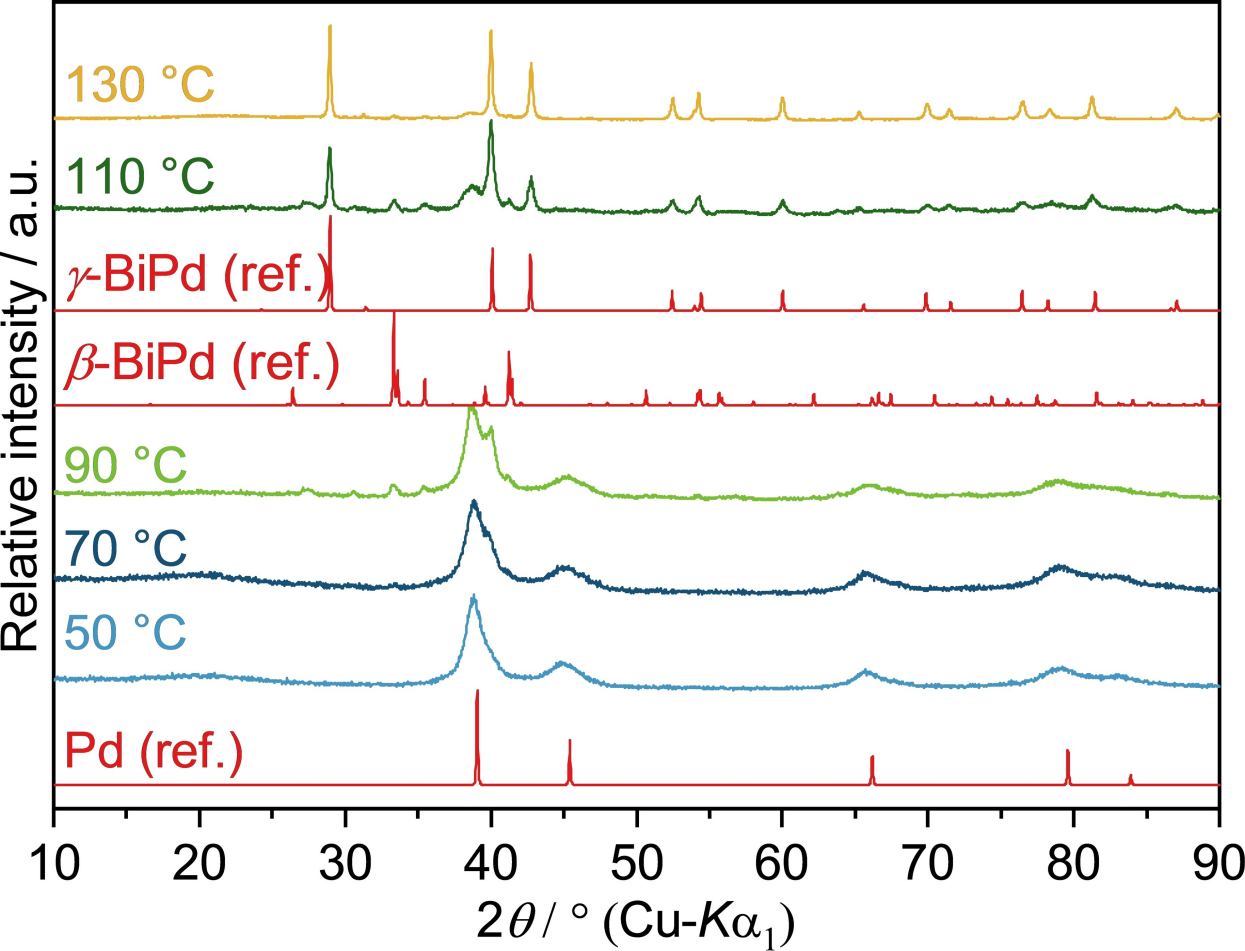
PXRD patterns of solid reaction products obtained from the reaction of Pd(OAc)_2_ and Bi(NO_3_)_3_ in alkaline EG at different temperatures after 10 min reaction time. Pd (ref.), *β*‐BiPd (ref.) and *γ*‐BiPd (ref.) are calculated diffraction patterns based on the crystal structures of Pd (ICSD‐64914), *β*‐BiPd (ICSD‐56279) and *γ‐*BiPd (ICSD‐108171), respectively.

The addition of a strong base does not only promote the reduction of metal cations in the polyol process,^[25]^ but also inhibits the precipitation of bismuth glycolates.^[20c]^ Therefore, the reaction of Pd(OAc)_2_ and Bi(NO_3_)_3_ was also performed with the addition of KOH (Pd^2+^ : OH^−^=1 : 40). As anticipated, the required reaction temperature was reduced by 90 °C with *γ‐*BiPd already precipitating at approx. 110 °C (Figure 5). At 130 °C, a nearly phase pure product was obtained with traces of other *β*‐BiPd. No signs of a bismuth glycolate precipitate were found in the powder patterns throughout the entire reaction sequence, indicating that bismuth cations remain in solution, available for interactions with palladium particles. SEM images displayed similar particle assemblies as the reactions without KOH (Figure S4a & b).

Pure Bi(NO_3_)_3_ in alkaline EG needs approx. 180 °C for a reduction to occur. The Bi^3+^ reduction is obviously promoted by the presence of a noble metal as pure Bi(NO_3_)_3_ in alkaline EG needs approx. 180 °C to be reduced. The same trend has been observed in the systems Bi−Rh and Bi−Ir.[^20a^, ^20c^] In order to understand, if elemental Pd particles or the Pd salt is crucial, we mimicked the reaction with freshly prepared palladium particles (for preparation see experimental section) with Bi(NO_3_)_3_ under alkaline conditions at 120 °C (Figure S5a). Indeed, a reduction of bismuth cations occurred, leading to the formation of mostly *α*‐BiPd and Bi_2_Pd. Additionally, small amounts of *γ*‐BiPd were found accompanied by minor traces of elemental bismuth. Naturally, the palladium particles, synthesized separately, isolated and washed before the Bi^3+^ reduction, display a different reaction behavior as the ones formed *in situ*, since particle size, agglomeration and surface chemistry vary. Furthermore, we also investigated the possibility of palladium particles reacting with bismuth particles (Figure S5b). The freshly prepared particles (see Experimental Section) of both metals were re‐dispersed in EG, and the suspension was heated to 220 °C for 15 min. This reaction yielded *γ*‐BiPd only in small amounts, accompanied by elemental palladium, traces of elemental bismuth and other Bi−Pd intermetallics, which highlights the complex interactions possible in these reactions.

**Figure 6:**
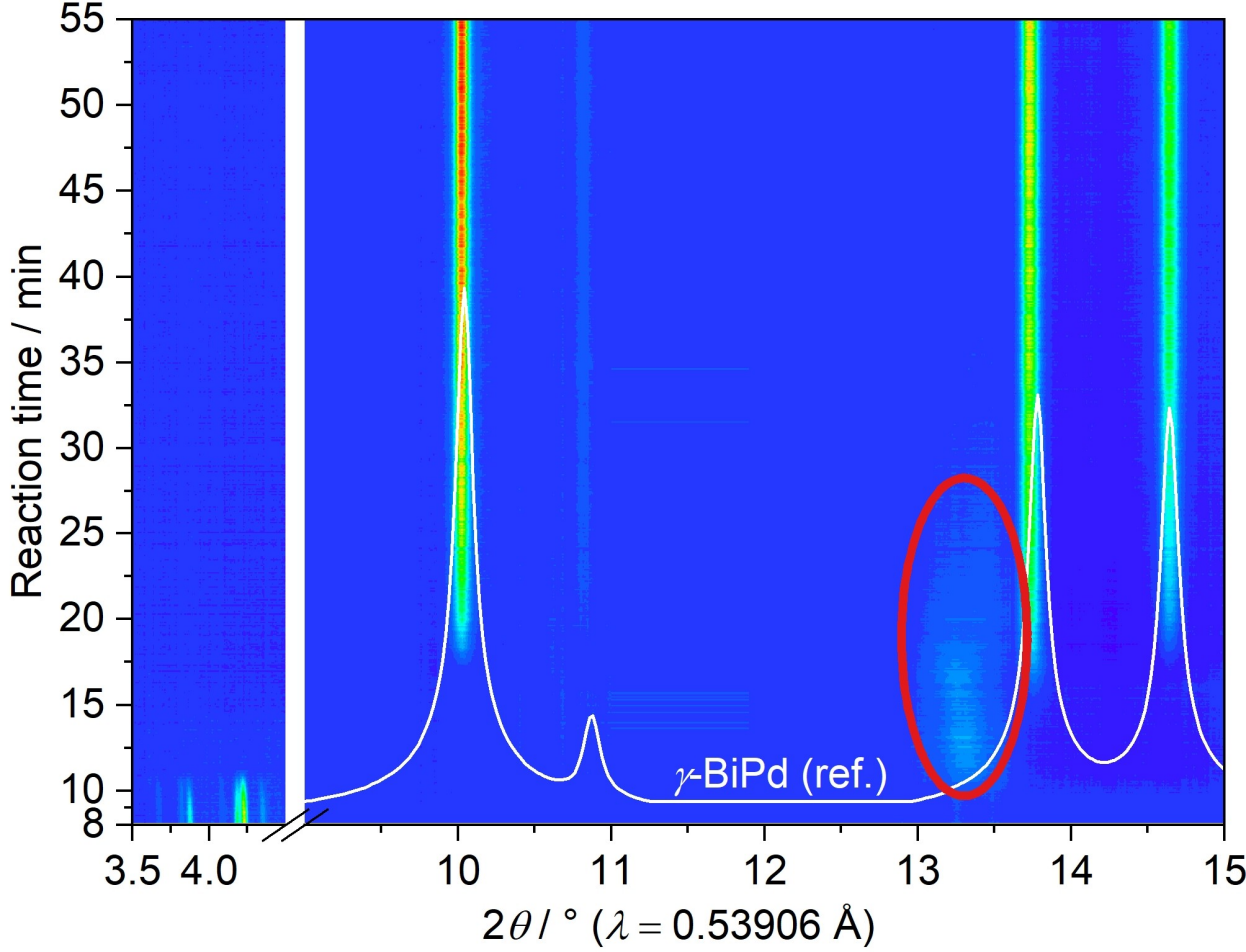
*In situ* PXRD measurement tracking the reaction of Pd(OAc)_2_ and Bi(NO_3_)_3_ in alkaline EG. The interval 4.5°≤2*θ*≤9° was excised due to a strong contribution of diffuse scattering by the glass vessel. *γ*‐BiPd (ref.) is the calculated diffraction pattern based on the crystal structure of *γ*‐BiPd (ICSD‐108171). The red ellipse marks the broad reflection of elemental palladium nanoparticles.

The reaction of Pd(OAc)_2_ and Bi(NO_3_)_3_ in alkaline EG was also monitored by *in situ* PXRD measurements (Figure 6). To enable a minimal pathway of the synchrotron X‐ray beam through the reaction volume, a modified glass reactor containing an inserted glass tube was applied (Figure S1). The reactor holder comprises an integrated stirring and heating system. In addition, it contains two orifices for the X‐ray beam inlet and outlet.^[26]^ At the beginning of the reaction, reflections of the insoluble Pd(OAc)_2_ are visible in the small‐angle range from 3.5°≤2*θ*≤4.5°. After ca. 11 min (at about 45 °C), a broad, diffuse reflection (red ellipse) is observed in the range of 13°≤2*θ*≤13.6°, which is in accordance with the broad reflections of palladium in the *ex situ* PXRD data (Figure 3). Simultaneously, the reflections of Pd(OAc)_2_ disappear, indicating the reduction of Pd^2+^ cations. After approx. 17 min (at about 85 °C), reflections of the *γ*‐BiPd phase emerge rather promptly and concurrently, as the reflection of elemental palladium decreases in intensity. In agreement with the *ex situ* measurements, no other phases, such as elemental bismuth, were observed, hinting towards a reduction of bismuth cations, probably on the surface of palladium particles, followed by a rapid diffusion and incorporation into the particle. Note that the maximum temperatures differ considerably between *in situ* and *ex situ* measurements.

### Co‐reduction of PdCl_2_ and Bi(NO_3_)_3_ in EG

**Figure 7:**
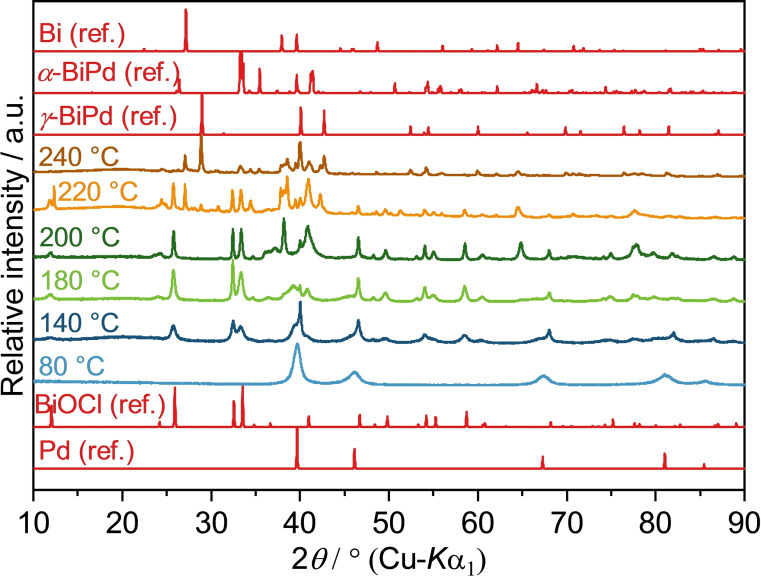
PXRD patterns of solid reaction products obtained from the reaction of PdCl_2_ and Bi(NO_3_)_3_ in neat EG at different temperatures after 10 min reaction time. Pd (ref.), Bi (ref.), BiOCl (ref.), *γ*‐BiPd (ref.) and *α*‐BiPd (ref.) are calculated diffraction patterns based on the crystal structures of Pd (ICSD‐257579), Bi (ICSD‐64703), BiOCl (ICSD‐195115), *γ*‐BiPd (ICSD‐108171) and *α*‐BiPd (ICSD‐54976), respectively.

In previous works we found that chloride ions pose an undesired effect on the reaction outcome due to the formation of the stable BiOCl.[^20a^, ^20c^] Hence, we also tested PdCl_2_ and its effects on the reaction. According to PXRD measurements (Figure 7), the reaction of PdCl_2_ and Bi(NO_3_)_3_ (Pd^2+^ : Bi^3+^=1 : 1) in neat EG yielded elemental palladium at 80 °C, which is consistent with the reduction temperature of a pure PdCl_2_ dispersion in EG. Not unexpectedly, the reaction resulted in the precipitation of BiOCl and palladium at 140 °C. Raising the temperature to 220 °C, reflections of elemental bismuth emerge through reduction of BiOCl, which are accompanied by minor traces of various Bi−Pd phases. The reflections visible in the region from 38°≤2*θ*≤41° in the samples obtained at 200 °C and 220 °C could not be assigned with certainty due to the overlap of multiple Bi−Pd phases. An increase of the temperature to 240 °C resulted in the formation of *γ*‐BiPd and traces of various Bi−Pd side phases again. Additionally, the intensity of bismuth reflections decreases to some extent, suggesting a partial incorporation into intermetallic species. The precipitation of BiOCl apparently led to a heterogeneous distribution of reactive species, resulting in a mixture of products. However, elemental bismuth apparently reacts with the existing particles to some extent.

**Figure 8:**
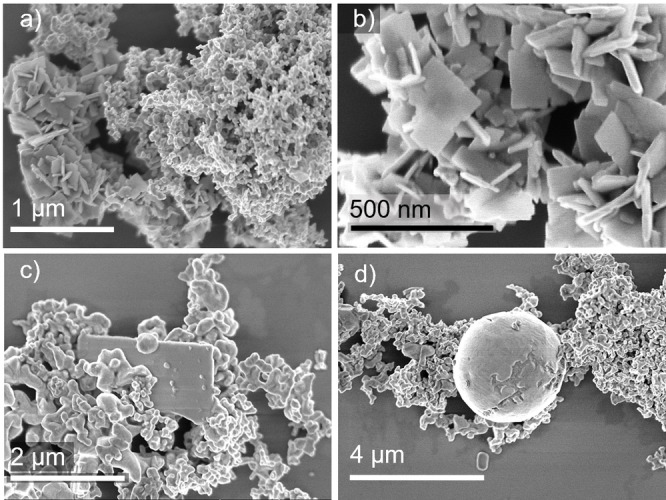
SEM images of particles obtained from the reaction of PdCl_2_ and Bi(NO_3_)_3_ in neat EG at (a, b) 180 °C and (c, d) 220 °C.

**Figure 9:**
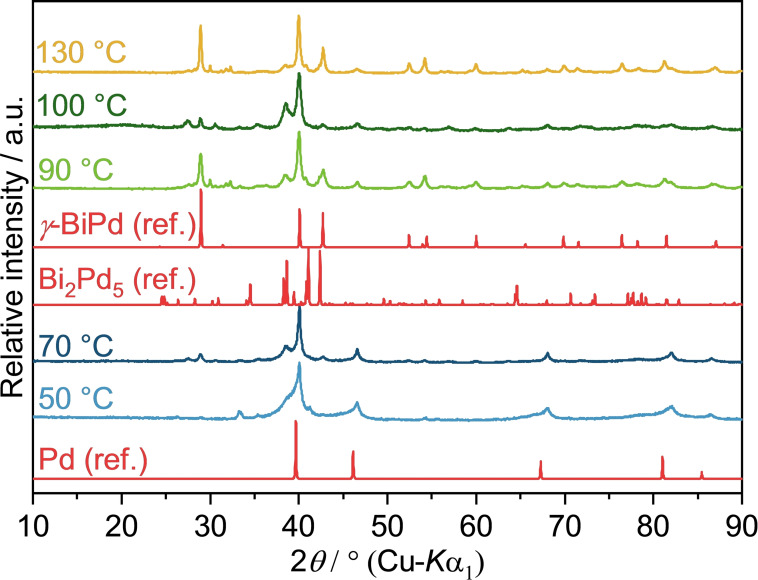
PXRD patterns of solid reaction products obtained from the reaction of PdCl_2_ and Bi(NO_3_)_3_ in alkaline EG at different temperatures after 10 min reaction time. Pd (ref.), Bi_2_Pd_5_ (ref.) and *γ*‐BiPd (ref.) are calculated diffraction patterns based on the crystal structures of Pd (ICSD‐257579), Bi_2_Pd_5_ (ICSD‐58840) and *γ*‐BiPd (ICSD‐108171), respectively.

Solid products of samples reacted at 180 °C and 220 °C were investigated by SEM (Figure 8). According to PXRD and EDS measurement, the sample isolated at 180 °C consisted of BiOCl elemental palladium, Figure 9a. The porous agglomerates of palladium particles (right part of Figure 8a) range from ca. 30 nm to 100 nm, BiOCl plates (left side of Figure 8a) are found with edge length of approx. 300–400 nm. Figure 8b shows a close‐up image of these platelets. EDS measurements confirmed the elemental ratio of 1 : 1 (Bi : Cl).

The sample obtained at 220 °C consisted of different Bi−Pd phases, BiOCl, and elemental bismuth. Figure 8c displays the intermetallic phases as agglomerates of particles exhibiting an average elemental ratio of 3 : 2 (Pd : Bi) as measured by EDS. A platelet of BiOCl with spherical particles on its surface, probably due to the reduction to elemental bismuth on the surface, is nested in between.^[20b]^ Figure 8d shows a similar image, but with a sphere of elemental bismuth with an approximate diameter of 3 μm is found between the intermetallic particles.

PXRD measurements of products obtained under alkaline conditions (addition of KOH, Pd^2+^ : OH^−^=1 : 40) evidence the formation of palladium at 50 °C, in agreement with a pure PdCl_2_ dispersion in alkaline EG, followed by *γ*‐BiPd formation between 70 and 90 °C (Figure 9). The target compound is again not obtained as a single‐phase product as reflections of traces of other Bi−Pd phases can be seen. The formation of BiOCl was not observed in this case. The enhanced complexation of bismuth cations by deprotonated EG probably leads to a retention of Bi^3+^ in solution, allowing for a homogeneous reaction with the palladium particles. Similar to reactions with Pd(OAc)_2_ under alkaline conditions, the required reduction temperature for bismuth cations is drastically lowered by 90 to 110 °C. SEM images of the precipitates isolated at 70 °C and 130 °C show agglomerates of particles ranging from 50–200 nm (Figure S7).

### Co‐reduction of K_2_[PdBr_4_] and Bi(NO_3_)_3_ in EG

**Figure 10:**
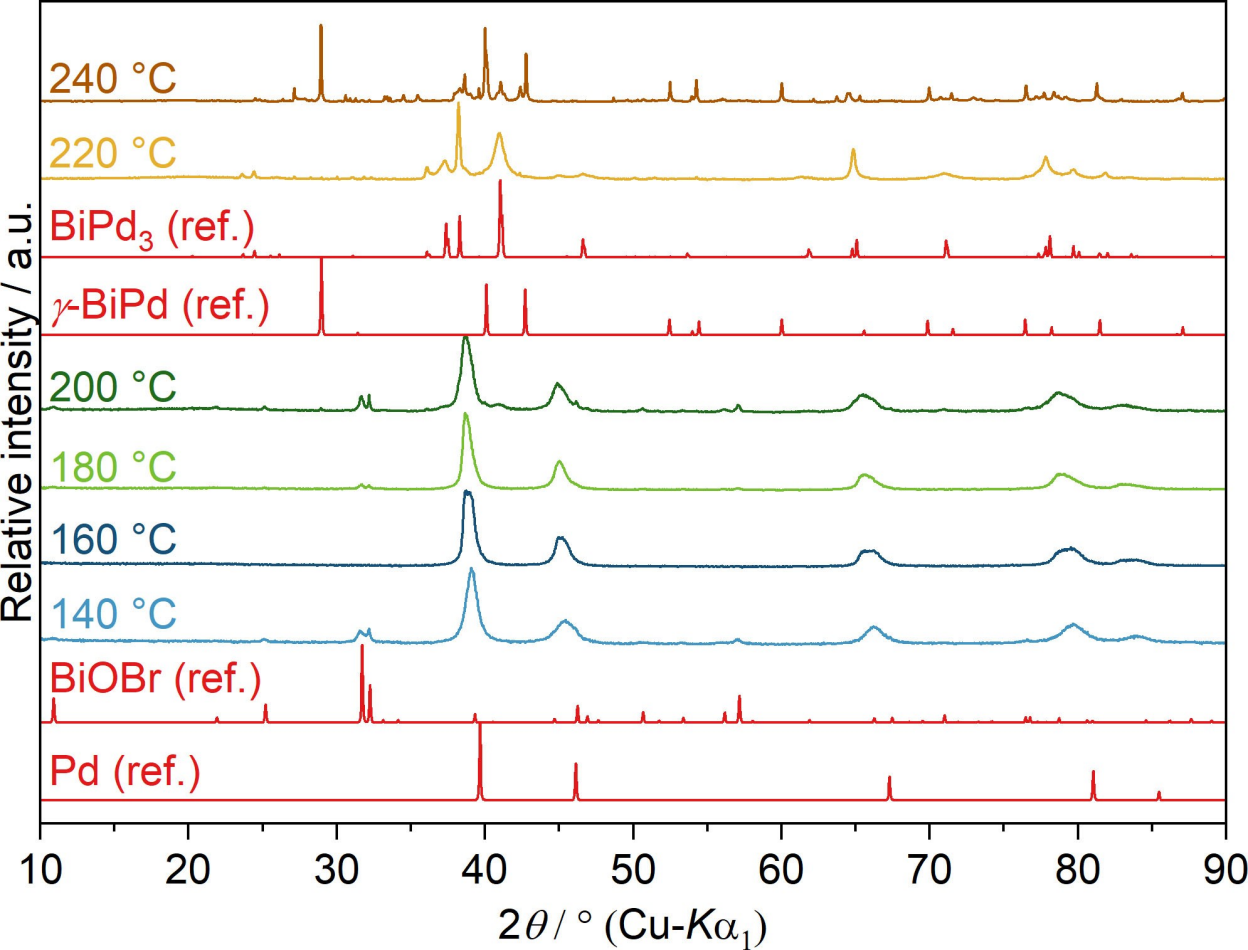
PXRD patterns of solid reaction products obtained from the reaction of K_2_[PdBr_4_] and Bi(NO_3_)_3_ in neat EG at different temperatures after 10 min reaction time. Pd (ref.), BiOBr (ref.), BiPd_3_ and *γ*‐BiPd (ref.) are calculated diffraction patterns based on the crystal structures of Pd (ICSD‐257579), BiOBr (ICSD‐ 61225), BiPd_3_ (ICSD‐ 58839) and *γ*‐BiPd (ICSD‐108171), respectively.

Last, we studied the reaction behavior of K_2_[PdBr_4_] and Bi(NO_3_)_3_. As in the previous cases, the first precipitate of the reaction of K_2_[PdBr_4_] with Bi(NO_3_)_3_ in neutral EG was identified as elemental palladium at approx. 130 °C; this is also the predominant phase up to reaction temperatures of about 220 °C (Figure 10). In contrast to reactions with PdCl_2_, only minor traces of BiOBr were identified, which is reasonable due to the lower nucleophilicity of bromide as compared to chloride.

In accordance with the temperatures required to reduce bismuth cations in neat EG (about 220 °C), a first palladium‐rich intermetallic in the form of BiPd_3_ was collected. Increasing the temperature further led to the formation of *γ*‐BiPd with traces of Bi, BiPd_3_ and other palladium‐rich intermetallic species.

**Figure 11:**
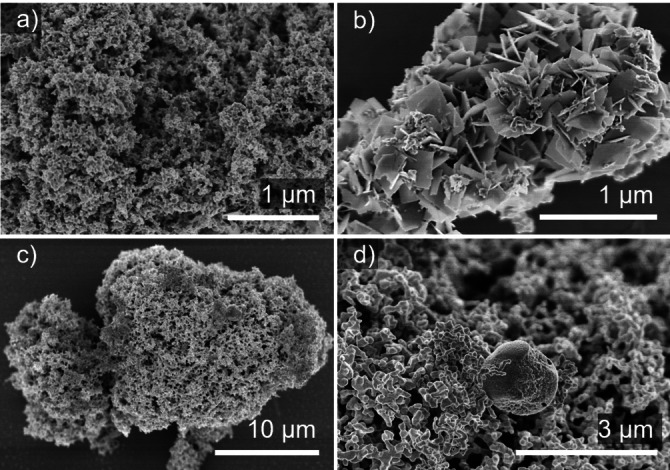
SEM images of particles obtained from the reaction of K_2_[PdBr_4_] and Bi(NO_3_)_3_ in neat EG at (a)&(b) 140 °C, and (c) & (d) 240 °C.

**Figure 12:**
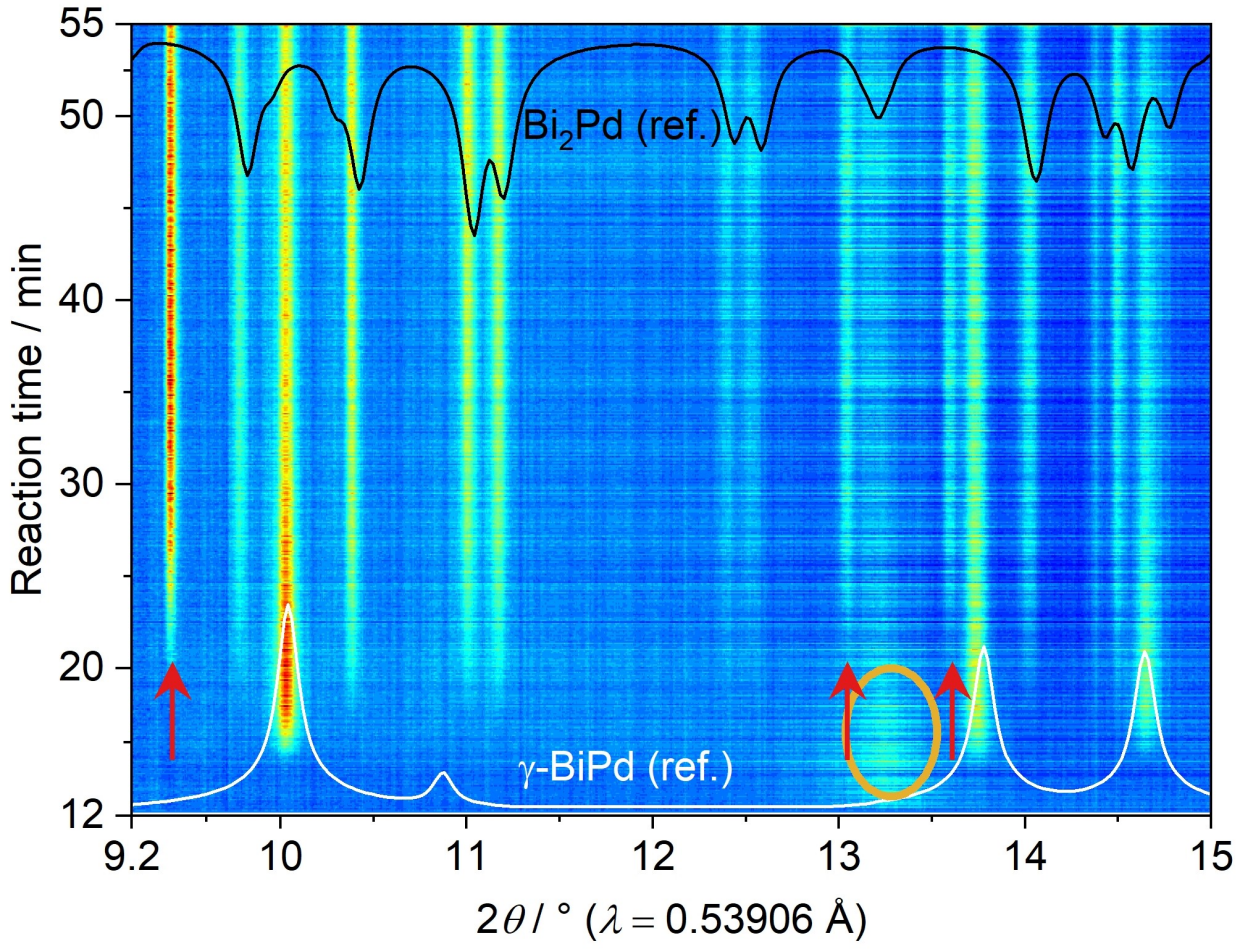
*In situ* PXRD measurement tracking the reaction of K_2_[PdBr_4_] and Bi(NO_3_)_3_ in alkaline EG. Bi_2_Pd (ref.) and *γ*‐BiPd (ref.) are the calculated diffraction patterns based on the crystal structures of Bi_2_Pd (ICSD‐42565) and *γ*‐BiPd (ICSD‐108171). The red arrows point towards the reflections of elemental bismuth based on the crystal structure (ICSD‐64703).

SEM images recorded at 140 °C display agglomerated palladium particles (Figure 11a). Additionally, BiOBr, which is isostructural to BiOCl, forms platelet‐like structures with edge lengths of 200–300 nm (Figure 11b). Increased reaction temperatures lead to a disappearance of the BiOBr plates and resulted in agglomerated particles of intermetallic Bi−Pd phases (Figure 12c). Sporadically, large, spherical particles of elemental bismuth with diameters of up to 1.5 μm were found (Figure 11d).

Reactions in alkaline medium, however, diverge completely from the aforementioned mechanisms. As stated above, K_2_[PdBr_4_] most likely reacts to K_2_[Pd(OH)_4_] in alkaline solutions, which is then reduced to elemental palladium at approx. 70 °C (Figure S8). In contrast to the other reactions in alkaline solution, the sample obtained at 130 °C consists predominantly of palladium with minor traces of *γ*‐BiPd. Raising the reaction temperature to 150 °C yielded a mixture of various phases, mainly elemental bismuth, Bi_2_Pd and Bi_3_Pd_8_. The broad spectrum of products does not change significantly with increasing temperatures; it is most like the consequence of a substantial agglomeration which occurs shortly after the precipitation of palladium, Figure S9. SEM images of the samples display agglomerated particles in a sponge‐like fashion throughout the entire reaction (Figures S10a–d), which probably hinders diffusion and the progress of the reaction.

*In situ* PXRD measurements the reaction of K_2_[PdBr_4_] and Bi(NO_3_) displayed a similar reaction scheme, Figure 12. Again, elemental palladium precipitates first, visible by a diffuse reflection in the range of 13°≤2*θ*≤13.6° (orange ellipse in Figure 12). After ca. 15 min (at about 95 °C), the formation of *γ*‐BiPd can be observed (Figure S11), which was also found in the *ex situ* PXRD measurements at 130 °C (Figure S8). Remarkably, after 20 min (at ~100 °C, i. e. the final reaction temperature), reflections of elemental bismuth appear (red arrows), which first emerged in the *ex situ* measurements between 140° and 150 °C.^[20c]^

Parallel to the bismuth formation, reflections of Bi_2_Pd emerge, while the intensity of *γ*‐BiPd reflections decreases. After roughly 45 min, the reflection intensities of Bi_2_Pd and *γ*‐BiPd do not change further. Reflections of Bi_3_Pd_8_, as observed in the *ex situ* measurements, are not present or are covered by overlapping reflections of the main products. Dissimilarities in intermediates and final products can be explained by variations in reaction and agglomeration rates caused by the different heating methods.

**Figure 13:**
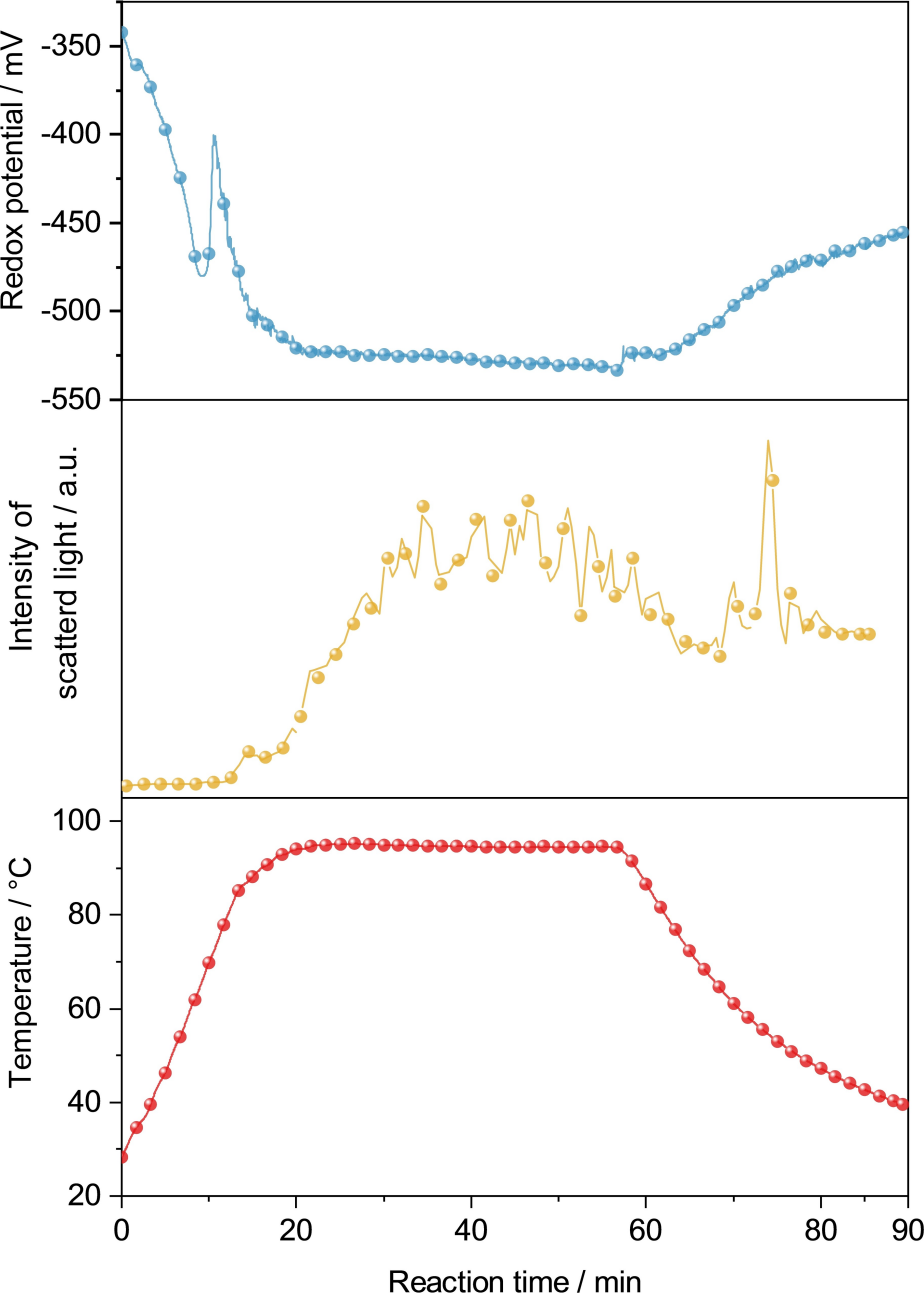
*In situ* measurements of redox potential (Ag/AgCl reference) and light scattering during the reaction of K_2_[PdBr_4_] and Bi(NO_3_)_3_ in alkaline EG.

In addition, *in situ* measurements of the redox potential and light scattering were performed for this reaction, too. Figure 13. In contrast to the measurements for the bimetallic reactions of Pd(OAc)_2_ and PdCl_2_ with Bi(NO_3_)_3_ in alkaline EG (Figures S12a&b) as well as the reduction of only K_2_[PdBr_4_] (Figure S2b), reactions of K_2_[PdBr_4_] with Bi(NO_3_)_3_ display a distinct peak in the redox potential indicating the formation of an oxidizing species, which decays over time. So far, it is unclear what produces this signal, but the solubility of K_2_[PdBr_4_] might give rise to additional redox reactions with the nitrate ions present in solution. Furthermore, the intensity of scattered light increases shortly after the intense signal indicating the growth of particles. However, the reaction temperature of 95 °C apparently does not suffice to prompt the substantial agglomeration observed in Figure S9, which would be noticeable by a drop in intensity of the scattered light.

## Summary

Systematic experiments were performed in order to study the formation mechanism of metastable *γ*‐BiPd particles in the polyol process and its dependency on temperature, pH value and palladium precursor. The main results of the *ex‐situ* investigations are compiled in Table 1.

**Table 1:** Results of the reduction of Bi(NO_3_)_3_ with different Pd sources in neat or alkaline EG. Primary products, further intermediates and the final products are listed together with their approximate temperatures of formation (*T*_prim_ and *T*_fin_) as identified by PXRD. Main products of the reactions are set in bold type.

Pd source	base	primary	*T*_prim_ [°C]	intermediates	final product(s)	*T*_fin_ [°C]
Pd(OAc)_2_	–	Pd	100	Bi‐gloycolate	***γ*‐BiPd**, BiPd_2_	240
Pd(OAc)_2_	KOH	Pd	50		***γ*‐BiPd**, *β*‐BiPd,	130
Pd NP	KOH				***α*‐BiPd**, Bi_2_Pd, *γ*‐BiPd	220
PdCl_2_	–	Pd	80	BiOCl	***γ*‐BiPd**, *α*‐BiPd, Bi	240
PdCl_2_	KOH	Pd	50		***γ*‐BiPd**, Bi_2_Pd_5_	130
K_2_[PdBr_4_]	–	Pd	140	BiOBr	***γ*‐BiPd**, BiPd_3_	240
K_2_[PdBr_4_]	KOH	Pd	70		**Bi**, **Bi_2_Pd**, Bi_3_Pd_8_, *γ*‐BiPd	230

Using Pd(OAc)_2_, the reaction begins with the precipitation of elemental palladium, before a solid bismuth glycolate is formed additionally at slightly elevated temperatures. After reaching about 200 °C, bismuth cations are reduced, probably at the surface of the palladium particles, followed by a rapid diffusion of Bi into the Pd nuclei and the formation of *γ*‐BiPd particle. A similar reaction pathway occurs when PdCl_2_ is used as starting material, however, with BiOCl as an intermediate. In the case of K_2_[PdBr_4_] as precursor, elemental palladium is again the first major solid precipitate accompanied by minor amounts of BiOBr. Reaching the reduction temperature of Bi^3+^ leads to the formation of BiPd_3_ as an intermediate phase followed by full conversion to *γ*‐BiPd. For Pd(OAc)_2_ as well as PdCl_2_, higher yields of *γ*‐BiPd and less by‐products were obtained upon addition of KOH, as the base prevents the precipitation of the intermediate bismuth glycolate and of BiOCl, respectively. In case of K_2_[PdBr_4_], the addition of KOH was not beneficial for the reaction but lead to considerable particle agglomeration in early reaction steps

## Conclusions

In the context of four different binary Bi‐containing intermetallic systems investigated under similar reaction conditions recently, we found some common trends but even more pronounced differences. In the Bi−Pd system presented here, the noble metal cations are reduced first and elemental Pd is always the primary reaction product in accordance with the electrochemical series. The Pd particles seem to promote the Bi^3+^ reduction and serve as nucleation sites for the subsequent formation of Bi−Pd phases by a solid‐state diffusion of elemental Bi into the Pd core. The reactions are noticeably facilitated by addition of a base and by increasing the temperature. Halide precursors, on the other hand, give rise to the formation of bismuth oxyhalides BiO*X* (*X*=Cl, Br) which are detrimental for the course of the reaction. The target compound *γ*‐BiPd was hardly obtained as a single‐phase product under the reaction conditions used in this work. In most cases, this can be attributed to the standardized short reaction time of 10 min. However, Heise has shown previously that phase pure products can be obtained in polyol reductions.^[7b]^ In reactions starting from K_2_[PdBr_4_], heavily agglomerated particles precipitate which additionally hinder the (probably diffusion controlled) course of the reaction.

The promoting effect of temperature and basic reactions conditions as well as the detrimental effect of halide ions were found for the systems Bi−Ni, Bi−Ir and Bi−Rh, too. However, much more differences in the reaction mechanisms of the four binary systems have been observed. The reduction of Bi^3+^/Ni^2+^ solutions proceeded via core‐shell particles with Ni nanoparticles in the shell of a Bi core, followed by a solid‐state diffusion towards the final product. In the Bi−Ir system, however, a new suboxide Bi_4_Ir_2_O was found as intermediate, pointing towards a simultaneous partial reduction of both metals. The suboxide is then fully reduced upon increasing the temperature. In the Bi−Rh case, a simultaneous co‐reduction of Bi^3+^ and Rh^2+^ was monitored within the experimental resolution despite the notable differences of the standard potentials.

As far as a tailored synthesis of (binary) intermetallic particles is aimed at, no general procedure can be proposed. Instead, most cases need to be studied separately and in detail to understand the specifics of the reaction mechanism and use this knowledge to develop specific synthesis conditions for maybe defined particle sizes, structures and properties.

## Experimental Section

### Chemicals

The following chemicals were used as without further purification: ethylene glycol (Honeywell, 99 %), HBr (Fluka Analytical, 48 %), Bi(NO_3_)_3_ ⋅ 5H_2_O (VWR, 98 %), Pd(OAc)_2_ (Sigma Aldrich, 99 %), PdCl_2_ (Sigma‐Aldrich, 99 %), K_2_[PdBr_4_] (abcr, 98 %). For some experiments, a 1 m solution of KOH (Fischer Chemicals, 99 %) in EG was used.

### Synthesis

Laboratory experiments were performed in an Anton Paar Monowave 400 synthesis microwave, using 30 mL vessels equipped with magnetic stirring bars that ensured intense mixing of the reagents. For each synthesis, the bismuth (0.1 mmol) and palladium (0.1 mmol) salts (amounts given with respect to the cations) were mixed with 10 mL of EG. In case of addition of 1 m KOH solution, 4 mL were added to 6 mL of EG. After dissolving/dispersing the starting materials, the reaction mixtures were heated by microwave radiation to the desired temperature within 10–30 s. At the end of the reaction time, the sample was cooled to 70 °C inside the microwave device with a stream of air and then removed. The precipitates were isolated by centrifugation at 4400 rpm, washed three times with ethanol (~15 mL) to remove residual solvent, and dried overnight under dynamic vacuum at room temperature.

For *in situ* measurements of the redox potential, pH and light scattering, 0.8 mmol of Pd(OAc)_2_, K_2_[PdBr_4_], or PdCl_2_ and 0.8 mmol Bi(NO_3_)_3_ were dispersed/dissolved in a mixture of 14 mL EG and 16 mL 1 m KOH. During the measurement, the solution was heated in a glass vessel (Figure S1) to ~105 °C under mild stirring. Synchrotron experiments were performed separately under the same conditions as the other *in situ* measurements. The time axes for the *in situ* diffraction measurements were shifted to account for the delay between mixing the components and the actual start of the experiment.

Palladium particles were prepared by dissolving 0.3 mmol of K_2_[PdBr_4_] in 10 mL of EG and heating the reaction mixture to 200 °C. The obtained particles were isolated and washed as described above. Afterwards, 0.1 mmol of said particles were dispersed in 6 mL of EG and 4 mmol of KOH and combined with 0.1 mmol of Bi(NO_3_)_3_. The reaction mixture was heated by microwave radiation to 120 °C for 15 min. The product was treated as describe above. Bismuth particles were synthesized by dissolving 0.5 mmol of Bi(NO_3_)_3_ in 10 mmol of EG. The reaction mixture was heated to 250 °C for 10 min and the product subjected to the washing procedure described above. Afterwards, 0.1 mmol of bismuth and palladium particles were dispersed in 10 mL of EG and the reaction mixture heated to 220 °C for 15 min. The precipitate was washed as described above.

### Characterization

PXRD patterns were recorded at 296(1) K with an X′Pert Pro MPD (PANalytical) or Empyrean (PANalytical) diffractometer equipped with a Johansson monochromator using Cu‐*K*α_1_ radiation (*λ*=1.54056 Å) and a Pixcel‐1D detector in Bragg–Brentano setup. The references in the powder diagrams have been calculated from single‐crystal structure data deposited in the ICSD.^[27]^

Synchrotron experiments were performed at the DESY Hamburg P23 beamline, using X‐rays with a wavelength of *λ*=0.53906 Å and a 2D detector (Teledyne DALSA Rad‐icon 2329, Canada). Powder diffraction patterns were recorded every five seconds. For the sake of clarity in the assignment of the *in situ* measured Bragg reflections to the respective simulated diffraction patterns, the XRD data was treated as suggested by Platero‐Prats et al. for the *in situ* total scattering measurements.^[28]^ Thus, our *in situ* XRD data was normalized to overcome the oscillation of the intensity of the synchrotron X‐ray beam. The background caused by the reactor walls and the solvent was subtracted.

UV‐Vis spectroscopy was performed in double beam mode on a Cary 50 (Varian) in standard silica cuvettes.

SEM images and EDS were recorded on a SU8020 (Hitachi) equipped with a triple detector system for secondary and low‐energy backscattered electrons applying an acceleration voltage of 2 keV for imaging and 20 keV for EDS (Oxford).

The redox potential, pH, and temperature were measured using a Metrohm Titrando dosing unit (Module 902, Metrohm AG, Switzerland) with pH sensor (JUMO GmbH, Germany), temperature sensor (Metrohm AG, Switzerland), and redox sensor (Jumo tecLine HD Rd single‐rod measuring cell, JUMO GmbH, Germany) being submersed in the reaction solution. The redox electrode is equipped with a glass shaft, solid gel, salt reservoir, platinum tip and PTFE diaphragm and operates in the range +/−1500 mV at temperatures between 0 °C and 135 °C and pressures up to 13 bar against; a pressure‐compensated double‐chamber system with a silver/silver chloride (Ag/AgCl) line system in cartridge form is used as a reference.

For the measurement of the *in situ* light scattering, an LED‐emitting light source (Sahlman Photochemical Solutions, Germany) with a wavelength of 365 nm was placed outside the reactor at an angle of 90°. The scattered light was detected by a fiber optic cable placed outside of the reactor. The fiber optic cable was connected to a mobile EPP20000 spectrometer (Stellar Net Inc., USA) equipped with a CCD‐based detector.

## Conflict of interests

The authors declare no conflict of interest.

1

## Supporting information

As a service to our authors and readers, this journal provides supporting information supplied by the authors. Such materials are peer reviewed and may be re‐organized for online delivery, but are not copy‐edited or typeset. Technical support issues arising from supporting information (other than missing files) should be addressed to the authors.

Supporting Information

## Data Availability

The data that support the findings of this study are available from the corresponding author upon reasonable request.
