# Supplementary material for: In situ Investigations of the Formation Mechanism of Metastable γ‐BiPd Nanoparticles in Polyol Reductions
Source: ChemistryOpen. 2023 Dec 13;13(6):e202300103. doi: 10.1002/open.202300103 (PMC11164024; doi:10.1002/open.202300103)
Supplement: Supplementary file 1 — Supporting Information [file OPEN-13-e202300103-s001.pdf]

ChemistryOpen

Supporting Information

In situ Investigations of the Formation Mechanism of Metastable γ-BiPd Nanoparticles in Polyol Reductions

Matthias Smuda, Noah Elsner, Jonas Ströh, Nicole Pienack, Rastko Radulovic, Azat Khadiev, Huayna Terraschke, Michael Ruck, and Thomas Doert*

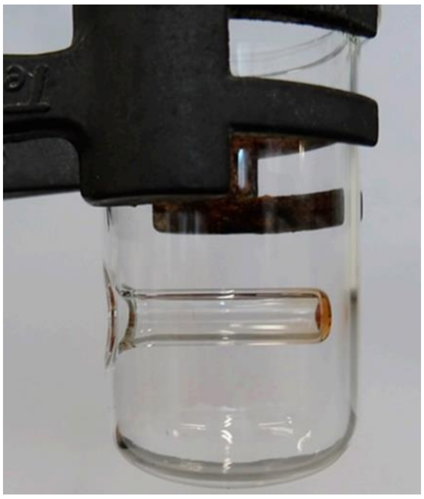

Figure S1. Reaction vessel with a glass tube fixed inside to minimize the pathway of the synchrotron X-ray beam through the reaction volume.

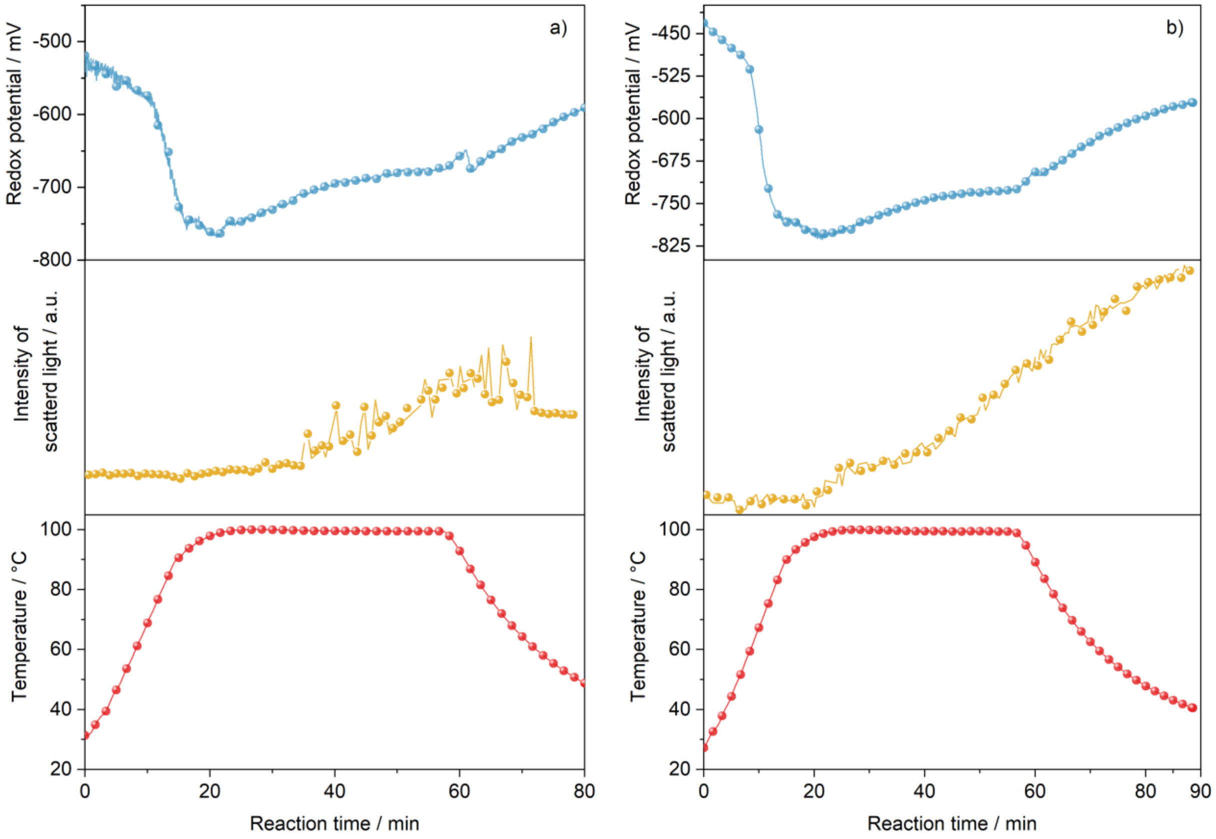

Figure S2. *In situ* measurements of the redox potential and light scattering during the reduction of a) $PdCl_2$ and b) $K_2[PdBr_4]$ in alkaline EG.

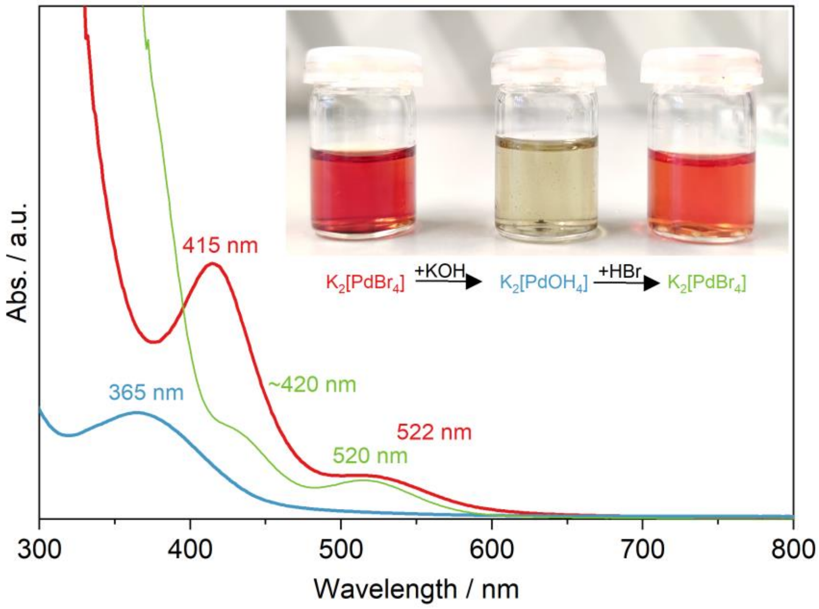

Figure S3. UV-Vis spectra of $K_2[PdBr_4]$ dissolved in neutral (red) and alkaline (blue) EG, and after acidifying with hydrobromic acid (green).

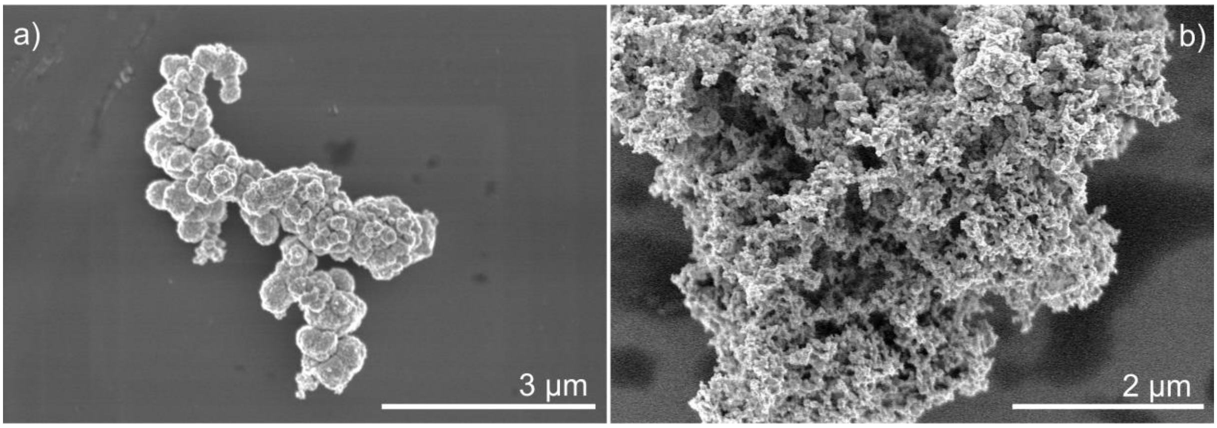

Figure S4. SEM images of particles obtained from the reaction of $Pd(OAc)_2$ and $Bi(NO_3)_3$ in alkaline EG at (a) 70 °C and (b) 130 °C.

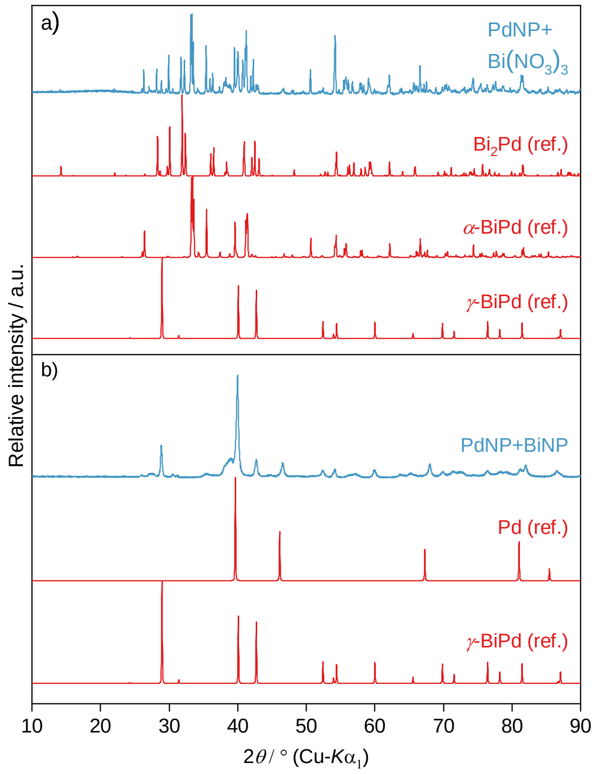

Figure S5. PXRD patterns of solid reaction products obtained from the reactions of a) palladium nanoparticles and $Bi(NO_3)_3$ at 120 °C in alkaline EG for 15 min and b) palladium nanoparticles and bismuth nanoparticles in neat EG at 220 °C for 15 min. Pd (ref.), *γ*-BiPd (ref.), *α*-BiPd (ref.), and Bi_2Pd (ref.) are calculated diffraction patterns based on the crystal structures of Pd (ICSD-64914), *γ*-BiPd (ICSD-108171), *α*-BiPd (ICSD-54976), and Bi_2Pd (ICSD-42565).

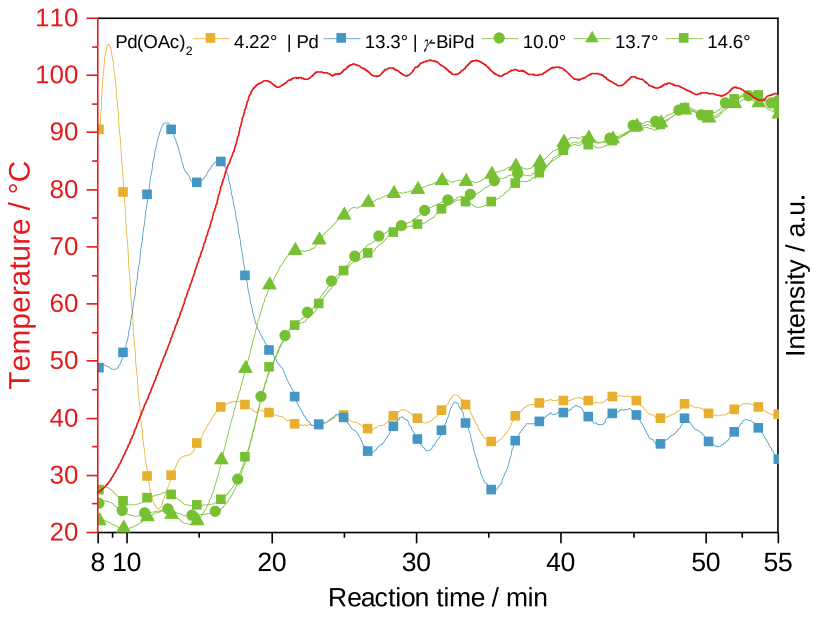

Figure S6. Evolution of diffraction intensities during the *in situ* PXRD measurement of $Pd(OAc)_2$ and $Bi(NO_3)_3$ for selected reflections of the three observed phases: $Pd(OAc)_2$ (orange), Pd (blue) and *γ*-BiPd (green). The red curve describes the temperature profile.

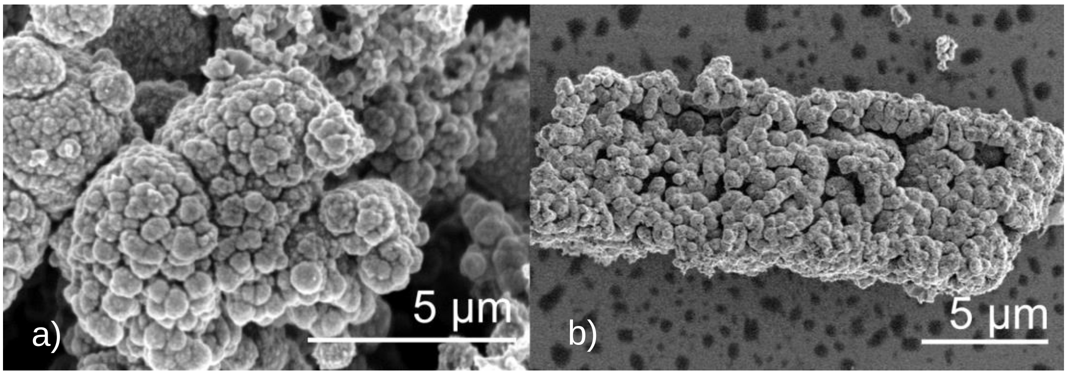

Figure S7. SEM images of particles obtained from the reaction of $PdCl_2$ and $Bi(NO_3)_3$ in alkaline EG at 70 °C (a) and 130 °C (b).

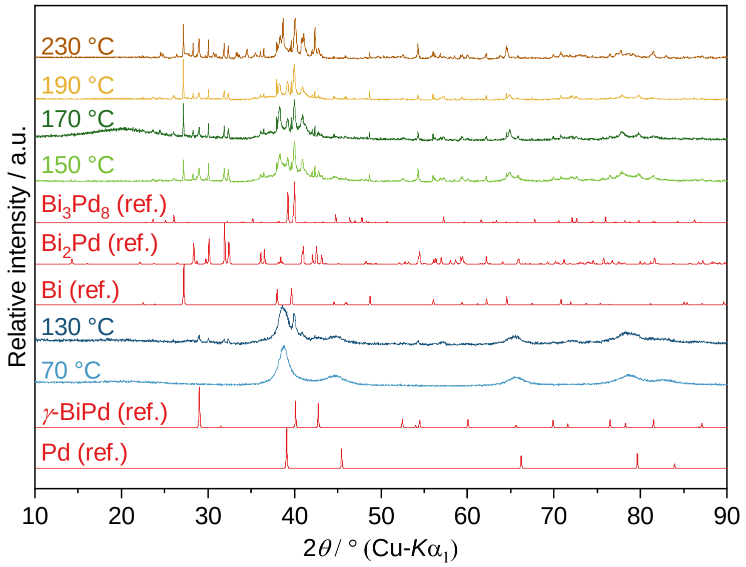

Figure S8. PXRD patterns of solid reaction products obtained from the reaction of $K_2[PdBr_4]$ and $Bi(NO_3)_3$ in alkaline EG at different temperatures after 10 min reaction time. Pd (ref.), Bi (ref.), Bi_3Pd_8 (ref.), Bi_2Pd (ref.) and *γ*-BiPd (ref.) are calculated diffraction patterns based on the crystal structures of Pd (ICSD-64914), Bi (ICSD- 61225), Bi_3Pd_8 (ICSD- 616947), Bi_2Pd (ICSD-42565) and *γ*-BiPd (ICSD-108171), respectively.

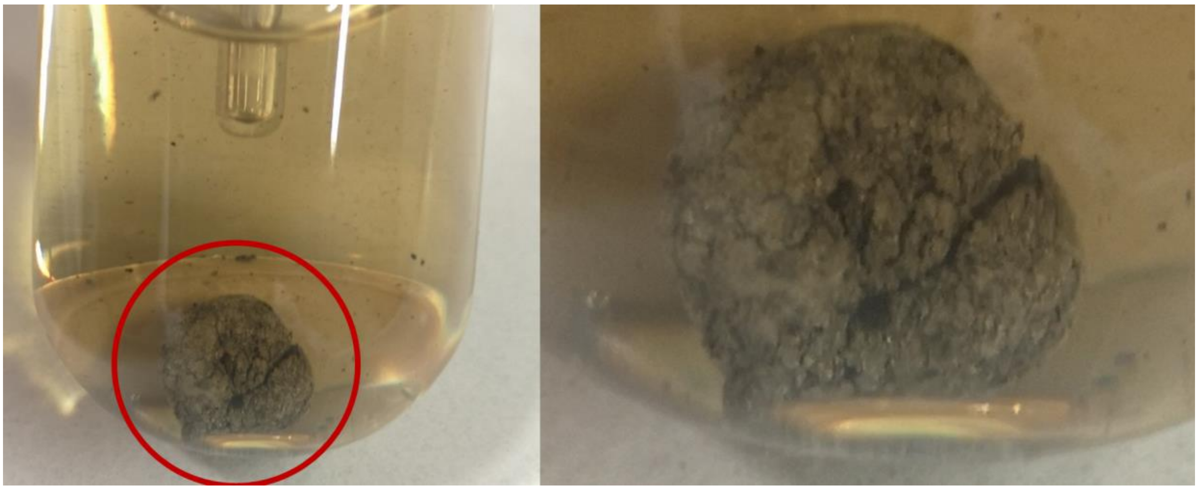

Figure S9. Photos of the severely agglomerated particles obtained from the reaction of $K_2[PdBr_4]$ and $Bi(NO_3)_3$ in alkaline EG after 10 min at 240 °C.

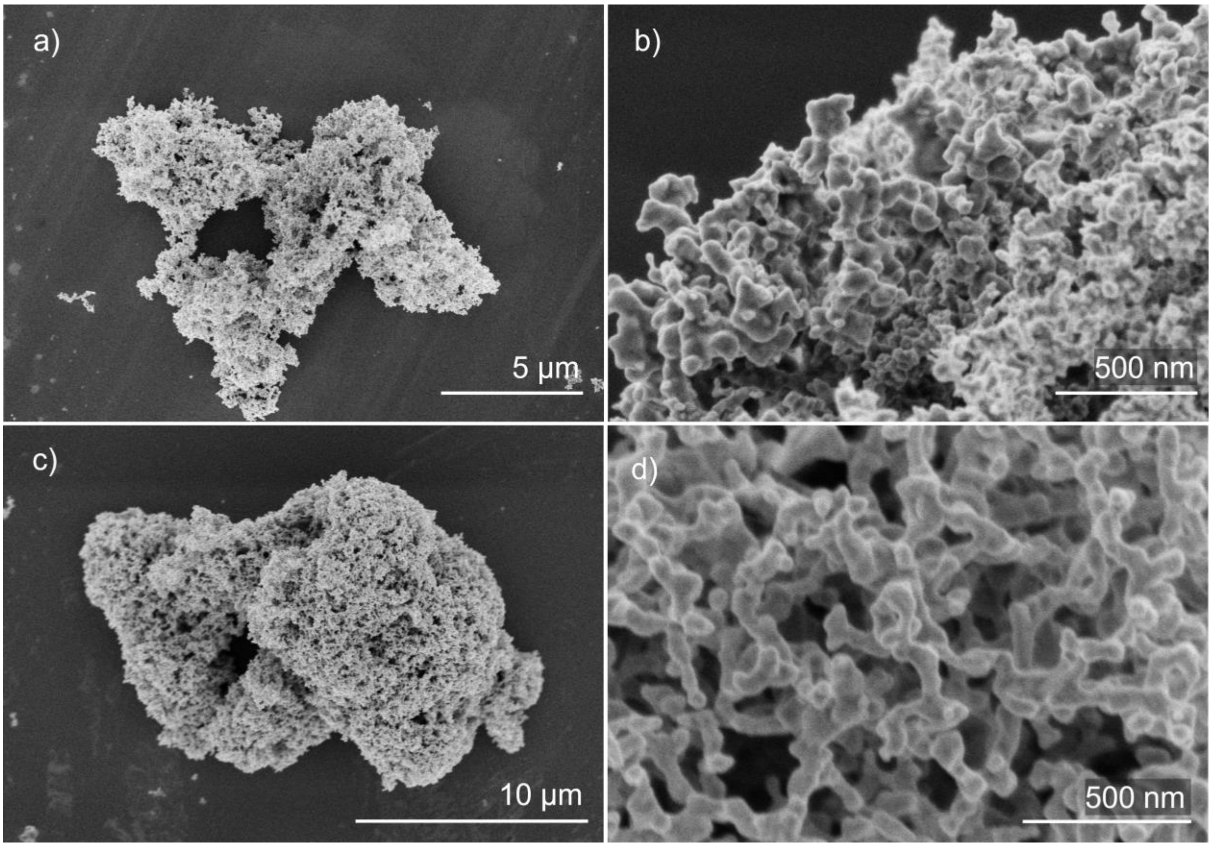

Figure S10. SEM images of particles obtained from the reaction of $K_2[PdBr_4]$ and $Bi(NO_3)_3$ in alkaline EG at 150 °C (a & b) and 230 °C (c & d).

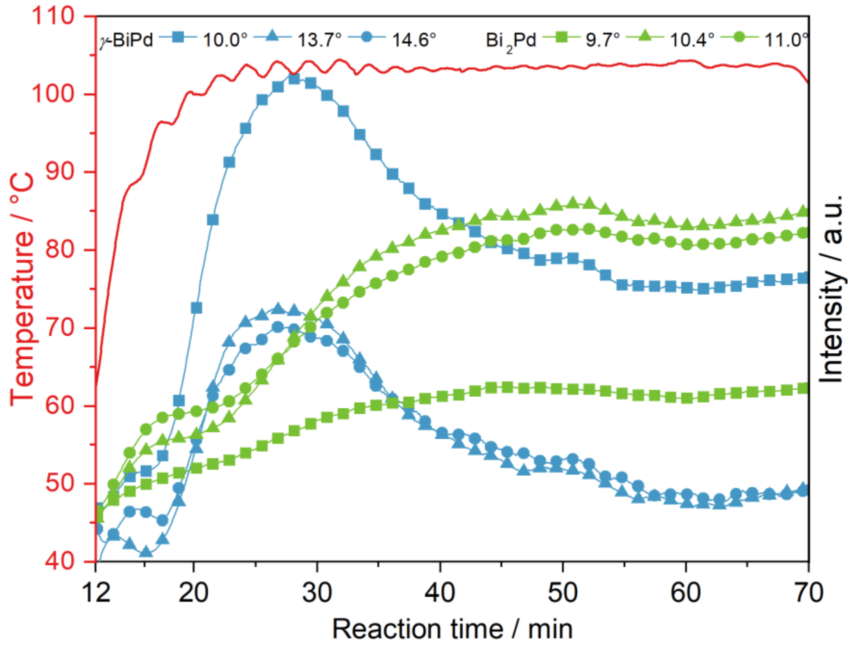

Figure S11. Evolution of diffraction intensities during the *in situ* PXRD measurement of $PdCl_2$ and $Bi(NO_3)_3$ for selected reflections of the two observed phases: Bi_2Pd (green) and *γ*-BiPd (blue). The red curve describes the temperature profile.

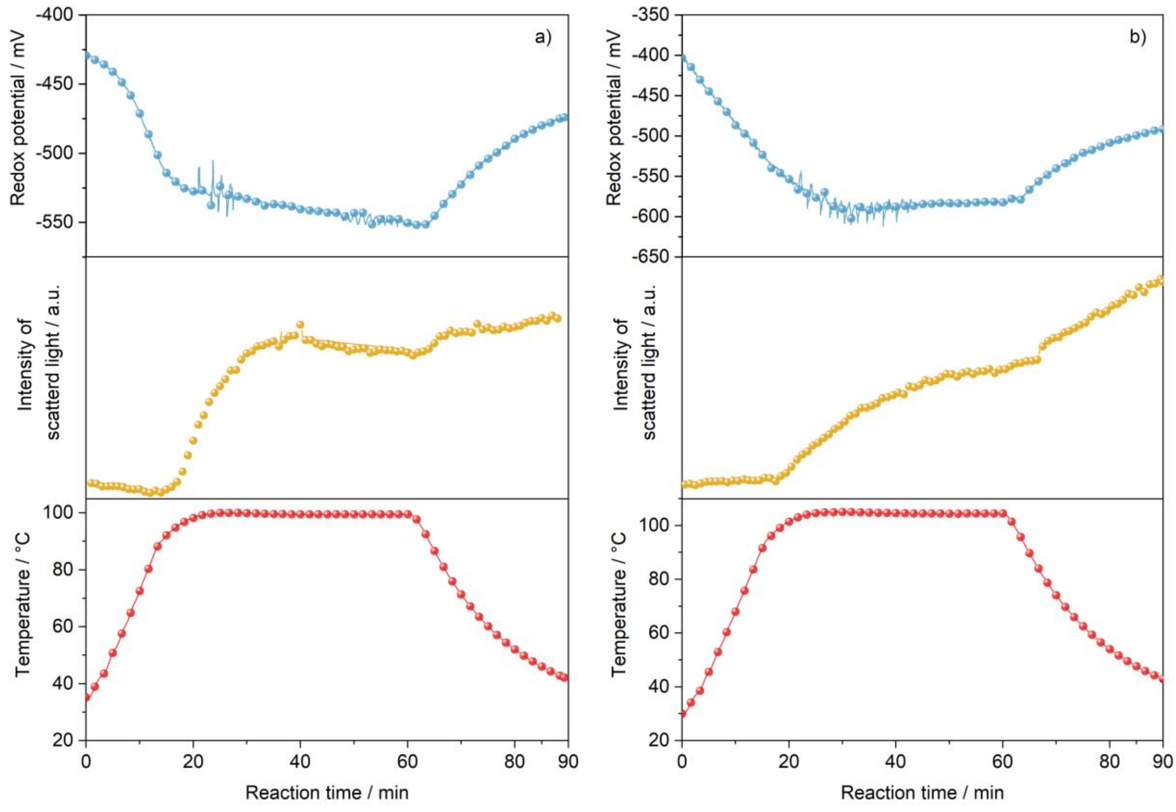

Figure 12. *In situ* measurements of the redox potential and light scattering during the reaction of a) $Pd(OAc)_2$ and b) $PdCl_2$ with $Bi(NO_3)_3$ in alkaline EG.
